# Multiple Mini-Interviews (MMI) and Semistructured Interviews for the Selection of Family Medicine Residents: A Comparative Analysis

**DOI:** 10.1155/2014/747168

**Published:** 2014-08-05

**Authors:** Marie Andrades, Seema Bhanji, Samreen Kausar, Fouad Majeed, Sheilla Pinjani

**Affiliations:** ^1^Faculty Family Medicine, Aga Khan University, Karachi 3500, Pakistan; ^2^Consultant Family Medicine, Prince Salman Armed Forces Hospital, Tabuk 71411, Saudi Arabia; ^3^Faculty Departement of Educational Development, Aga Khan University, Karachi 74800, Pakistan

## Abstract

*Background*. Family Medicine Residency Program at the Aga Khan University has applicants for the residency position in excess of the positions offered resulting in formulation of certain selection criteria. The objective of this study was to compare MMI versus semistructured interviews for assessing noncognitive domains in the selection of residents. The secondary objectives were to determine perceptions of the interviewers and candidates for the acceptability and feasibility of MMI as a selection tool. *Methods*. The candidates underwent semistructured interviews along with MMI and identical attributes were tested in both. The attributes tested were safe doctor, communication skills, professionalism, problem solving, team approach, ethical issues, reasons for selecting family medicine, and commitment to the program. Descriptive statistics were calculated and comparison between ratings for MMI and interview was performed by Wilcoxon sign rank test. *Results*. Total number of candidates was 14. On comparison between interview and MMI, the scores were not statistically different for all attributes except ethics (mean interview scores: 3.04, mean MMI scores: 2.5, and *P* value 0.046). *Conclusion*. The study showed no difference between MMI and semistructured interviews. However, it needs to be replicated in order to determine the predictive validity and feasibility of MMI over time.

## 1. Background

Globally residency positions offered are generally limited as compared to the number of candidates applying resulting in certain selection criteria [1]. Hence efforts are made to ensure that selection processes are fair and merit based, with reliable/valid/objective and standardized tools.

Currently several admission strategies have been adopted in residency programs [2]. The applicants' cognitive (academic) achievements are evaluated by written examinations and aptitude tests. The noncognitive domains like motivation and attributes of professionalism are assessed through interviews [1, 3]. However challenges to reliability and validity of interviews remain due to lack of training, structuring, and variation in scoring [4, 5].

The MMI is an internationally validated tool which comprises multiple station interviews with one or two interviewers rating candidates' responses. MMI has demonstrated evidence for generalizability and validity in relation to future clinical and licensing examination performance as compared to traditional interview methods. It has been used to measure professionalism for international graduates in residency selection at University of Calgary [6, 7]. In addition MMI has established acceptability with stakeholder groups at the admission level for both undergraduates and postgraduates [8, 9].

Family Medicine Residency Program at Aga Khan University (AKU) has the distinction of being the first residency-training program in family medicine in Pakistan [10]. Since inception the Residency Selection Committee (RSC) has used a semistructured interview format. An internal review of the residency program recommended incorporating a tool to better assess the noncognitive domains which may improve the selection process particularly as issues of professionalism were arising among the residents.

The objective of this study was to compare MMI versus semistructured interviews for assessing noncognitive domains in the selection of Family Medicine residents (Table 1).

The secondary objectives were to determine perceptions of the interviewers and candidates for the acceptability and feasibility of MMI as a selection tool.

## 2. Methods

### 2.1. Current Selection Process

Family Medicine Residency at Aga Khan University has a two tiered process for selection in which the cognitive domain is assessed through a written test. Those candidates shortlisted from the written test are then assessed for noncognitive attributes by an interview process. An average of 40–50 residency applicants competing for six positions sit for the test and about 15–18 applicants are shortlisted for the semistructured interviews.

The interview process consists of two separate panels of interviewers comprising three Family Medicine Faculty members in each panel. Each candidate is being interviewed by both the panels. The interview is semistructured (the questions are identical case scenarios for both panels; however there is no uniform accepted answer option). The attributes tested are safe doctor, communication skills, professionalism, problem solving, team approach, ethical issues, reasons for selecting Family Medicine, and commitment to the program. Each panel interview is of 20-minute duration in which a seven-point Likert scale is used by each faculty for scoring (see Table 3).

In 2010 we had a total of 49 candidate applications. Forty-seven appeared for the test and the first 16 highest scorers were shortlisted for the semistructured interview and MMI. As this MMI was a pilot selection method, the candidates were informed that they would be selected based on the interview scores and not MMI. Approval for the study was obtained from Ethical Review Committee of AKU.

The same attributes tested in interviews were used to develop the MMI stations. Eight stations were developed comprising situations the applicant would most likely face in a Family Medicine Residency (sample station Appendix B). Each station lasted for seven minutes and was designed to measure single or two to three of the attributes (mentioned before). Interviews were expected to rate on a Likert scale from 1–7 with 1 being poor and 7 being outstanding [11, 12]. Face validity of the stations was ensured through prior discussion with Family Medicine Faculty. MMI was conducted on the day following the semistructured interviews. A total of 16 candidates appeared for the interviews and 14 for the MMI. Two candidates regretted due to personal reasons. Each candidate rotated through the circuit comprising eight stations with 7 minutes per station adding to a total of 56 minutes per candidate in the circuit and was evaluated by a different interviewer at each station. Two circuits were run to accommodate the 16 candidates. The interviewers included senior Family Medicine Faculty residents and from the Department of Family Medicine and Educational Development (DED). Interviewers were trained through a combined training session conducted by the Family Medicine Department and DED. Immediate debriefing through structured questionnaires was obtained from the candidates regarding acceptability and from the interviewers for feasibility and acceptability after the MMI. Briefing about MMI was given to the candidates before the start of circuit. The clinical scenario based questions were pasted in at each station and candidates were required to read the question before beginning each station.

Candidate's feedback regarding MMI was assessed by a questionnaire using a seven-point Likert rating scale (Table 2). The questionnaire included the ability to portray themselves accurately, level of anxiety as compared to interviews, adequacy of pre-MMI instructions, need for specific knowledge for the stations, difficulty level of stations, time allocation for the stations, and reliability of the process. Similarly interviewer's feedback after the MMI was taken using the same attributes as above with the addition of feasibility in administration of interviews versus MMI and their opinion about replacing interviews with MMI. Descriptive statistics were calculated and comparison between ratings for MMI and interviews was performed by means of Wilcoxon sign rank test. The candidates and interviewers feedback was analyzed as frequencies.

## 3. Results 

Demographic background: a total of 16 candidates (12 female) sat in the interviews and a total of 14 sat in the MMI (11 female). Comparison was conducted for the 14 candidates who sat in both the tests. Eight out of 14 candidates had graduated within the last three years. Seven candidates have secured >70% marks in their final MBBS examination.

*Interviewers' Responses to Post-MMI Survey*. A total of eight interviewers were surveyed. Majority (87%) of the interviewers believed that they were able to get an accurate portrayal of the candidates. Fifty percent (4 out of 8) interviewers were unsure of the feasibility of conducting an MMI compared to the interviews. All but one of the interviewers thought that interviews can be replaced by MMI. The open narrative comments by faculty members included need for sound proof venue for the MMI stations and a post hoc analysis of the process.

## 4. Discussion

This studydoes notdemonstrate a statistical difference between MMI and semistructured interviews. A plausible explanation could be the type of questions in semistructured interview and examiner training could be one reason. Literature also supports the reliability of semistructured interviews [13]. Ethics was the only domain where the scores for MMI were less than that of semistructured interviews. A possible reason for this could be the large number of interviewers in the semistructured interview leading to a greater exploration of the subject. The results of this study are similar to a study done in United Kingdom where no difference was found between MMI and interviews for undergraduate applicants. MMI was found to be reliable, feasible, and acceptable to all the stakeholders [14]. Our experience of conducting the MMI was time and resource intense compared to interviews. A greater number of faculty and administrative staff were required for the MMI and a large venue was a challenge to obtain. Reviewing literature the same experiences is shared by others [15, 16].

Majority of the candidates felt they were being portrayed well through MMI. Most did not experience added anxiety during MMI versus the interview. MMI was considered to be a reliable selection tool by the candidates. Most were of the opinion that specific knowledge related to the attribute is required for each station. Other studies have also shown that participants have found MMI to be a positive experience [4, 17, 18]. Most interviewers felt that MMI can replace semistructured interviews as it accurately reflected the candidate's abilities [19].

Limitations of this study include a small sample size. In addition we had eight stations rather than the minimum of ten which was not possible because of limited availability of resources.

## 5. Conclusion

The purpose of conducting MMI is to select candidates who have the most suited desirable attributes for Family Medicine Residency Program. Based on this study there was no difference between semistructured interviews and MMI. Hence it is expected for the program to continue with semistructured interviews as MMI is more resource intensive. However, this study needs to be done longitudinally over time in order to have a better idea of its reliability and predictive validity.

## Figures and Tables

**Table 1:** Comparison of ratings of semistructured interviews and MMI stations (*n* = 14).

Serial number		Mean score semistructured interview	Mean score MMI	*P* value
1	Safe doctor	3.07	3.00	0.78
2	Communication skills	3.07	3.36	0.41
3	Problem solving	3.01	2.71	0.21
4	Professionalism	3.11	3.14	0.75
**5**	**Ethics**	**3.04**	**2.5**	**0.046**
6	Team member	3.02	2.93	0.84
7	Commitment to completing residency	2.75	3.07	0.21
8	Reasons for doing Family Medicine	2.64	2.64	0.92

**Table 2:** Frequencies of candidate's feedback response of MMI.

Serial number	Attributes	None	Somewhat					A lot
		1	2	3	4	5	6	7
1	Ability to portray themselves accurately	—	—	—	1	3	7	3
2	Anxiety during the selection process	5	3	1	—	4	1	—
3	Specialized knowledge needed for the stations	1	2	3	1	5	2	—
4	Reliability of selection method	—	—	2	3	2	5	2
5	Difficulty of exam	1	2	1	6	3	1	—
6	Adequate time allocation	—	—	—	4	8	1	1

Scoring by number of candidates (*n* = 14).

**Table 3:** Candidate interview evaluation form.

Candidate name ………		Date: ………						
Serial number	Attributes	Poor	Adequate				Outstanding	
		1	2	3	4	5	6	7
1	*Safe doctor*: knows his own limits, readily consults senior, cautious in taking risky decisions, and so forth.							

2	*Communication skills*: able to communicate clearly with colleagues, patients, and families							

3	*Problem solving skills*: logical and systematic in approach to clinical problems							

4	*Professional attitude*: empathic and compassionate towards patients and families: courteous towards all colleagues							

5	*Ethical*: sensitive towards confidentiality, patient rights, and moral values							

6	*Team member/interpersonal relationships*: efficient team member and utilizes input from other colleague							

7	*Reason for choosing Family Medicine: *							

8	*Commitment*: commitment to completing residency and the profession							

9	*General comments*: ………							

**Table 4:** 

Level	Points	Underlying scoring criteria
Outstanding	7	(i) Identifies it as a team problem and professor as team-leader (ii) Should be discussed within the team but maintains confidentiality by not discussing it outside the team. (iii) Would have owned up to the professor or senior registrar (iv) Reflects and learns from the situation by modifying approach (e.g., changing personal practice like counting instruments after surgery and educating other health care team members or teaching session on common errors in surgical practice) (v) The senior registrar or professor should communicate to the patient's family

Excellent	6	(i) Identifies it as a team problem and professor as team-leader (ii) Should be discussed within the team but maintains confidentiality by not discussing it outside the team. (iii) Would have owned up to the professor or senior registrar (iv) Reflects on the situation but does not identify the learning needs (v) The senior registrar or professor should communicate to the patient's family

Good	5	(i) Identifies it as a team problem and professor as team-leader (ii) Should be discussed within the team but maintains confidentiality by not discussing it outside the team. (iii) Would have owned up to the professor or senior registrar (iv) Fails to reflect on the situation (v) The senior registrar or professor should communicate to the patient's family

Adequate	4	(i) Identifies it as a team problem and professor as team-leader (ii) Does not discuss within the team but maintains confidentiality by not discussing it outside the team. (iii) Would have owned up to the professor or senior registrar (iv) Reflects on the situation but does not identify the learning needs (v) The senior registrar or Professor should communicate to the patient's family

Marginal	3	(i) Does not identifies it as a team problem and professor as team-leader (ii) Does not discuss within the team but maintains confidentiality by not discussing it outside the team. (iii) Would have owned up to the professor or senior registrar (iv) Does not reflect on the situation but does not identify the learning needs (v) The senior registrar or professor should communicate to the patient's family

Inadequate	2	Identifies only one or two criteria

Poor	1	Identifies no criteria

## Appendices

### A.

See Table 3.

### B. Sample Multiple Mini-Interview (MMI) Station: Testing Professionalism

Some time ago, there was* breaking news* of a professor being sacked/fired because of a death of a patient after a pair of scissors was left inside the peritoneum by a senior registrar during laparotomy, while a resident and nurse technician were assisting.

1. Who do you think was responsible?
2. Should it be communicated to other team members and hospital staff?
3. What would be your response, had you been assisting the senior registrar and left the scissors inside?
4. Having learned from this situation, what would you do in the future?
5. In your opinion who should talk to the patient's family?

Attributes are professionalism: ethics/confidentiality, teamwork, and reflection.

Most important attribute in this case is professionalism.

*Instructions for Interviewers*. This is a station where you will have to explore the attributes of professionalism, from the candidate. Let the candidate speak first and go through the scenario.

*Key*

*Positives*

- Identifies it as a team problem and professor as the team-leader
- Should be discussed within the team but maintains confidentiality by not discussing it outside the team.
- Would have owned up to the professor or senior registrar
- Reflects and learns from the situation by modifying their approach (e.g., changing personal practice like counting instruments after surgery and educating other health care team members or teaching session on common errors in surgical practice),
- Maybe the senior registrar or the professor should communicate to the patient's family.

*Negatives*

- Discusses the situation with the family him/her self
- Blames one or everyone responsible for the incident
- Does not want to communicate with the team
- Has insufficient insight of the implications of the problem

*Marking Key (see Table 4)*

*Expectations*. A candidate should have situation awareness (gravity of the situation), understanding of team responsibility, ability to reflect, propose solutions as change in practice (evidence of initiative), and maintains confidentiality.
Indicators for this domain include
  - understanding the needs of communication within team and family
  - understanding of responsibility as a team member
  - reflections on implications of critical incident on self and change in practice
  - implications of maintaining confidentiality and its breach.
